# Supplementary material for: Whole genome sequencing of hepatitis B virus using tiled amplicon (HEPTILE) and probe based enrichment on Illumina and Nanopore platforms
Source: Sci Rep. 2025 Feb 17;15:5795. doi: 10.1038/s41598-025-87721-1 (PMC11832747; doi:10.1038/s41598-025-87721-1)
Supplement: Supplementary file 3 — Supplementary Material 3 [file 41598_2025_87721_MOESM3_ESM.pdf]

Oct 16, 2024 Version 2

HEP-TILE: HBV whole genome sequencing (nanopore protocol) V.2

Version 1 is forked from ARTIC SARS-CoV-2 sequencing protocol v4 (LSK114)

DOI
dx.doi.org/10.17504/protocols.io.5jyl82bedl2w/v2

Sheila Lumley[1], Josh Quick[2], Philippa Matthews[3], Chris Kent[4]

[1]University of Oxford; [2]University of Birmingham; [3]The Francis Crick Institute; [4]ARTIC Network

HBV sequencing

Sheila Lumley
University of Oxford

OPEN ACCESS

DOI: dx.doi.org/10.17504/protocols.io.5jyl82bedl2w/v2

Protocol Citation: Sheila Lumley, Josh Quick, Philippa Matthews, Chris Kent 2024. HEP-TILE: HBV whole genome sequencing (nanopore protocol). **protocols.io** **https://dx.doi.org/10.17504/protocols.io.5jyl82bedl2w/v2**Version created by **Sheila Lumley**

Manuscript citation:
Whole genome sequencing of hepatitis B virus (HBV) using tiled amplicon (HEP-TILE) and probe-based enrichment on Illumina and Nanopore platforms. Lumley et al. 2024 https://www.medrxiv.org/content/10.1101/2024.09.11.24313306v1

License: This is an open access protocol distributed under the terms of the **Creative Commons Attribution License**, which permits unrestricted use, distribution, and reproduction in any medium, provided the original author and source are credited

Protocol status: Working
We use this protocol and it's working

Created: April 09, 2024

Last Modified: October 16, 2024

Protocol Integer ID: 110012

Keywords: hepatitis b virus, whole genome sequencing, HBV, tiled amplicon, nanopore sequencing

Funders Acknowledgement:
Wellcome trust
Grant ID: 102176/B/13/Z

Abstract

This protocol describes the HEP-TILE tiled amplicon protocol for whole genome sequencing of Hepatitis B virus (HBV) on the nanopore MinION.
We developed a pan-genotypic (genotypes A-J) HBV scheme using an early version of PrimalScheme3, a web-based primer design tool for developing multiplex primer schemes. PrimalScheme3 is a successor to PrimalScheme, with a number of changes made to enable us to generate an overlapping (tiled) amplicon scheme which covered the circular HBV genome, utilising a number of discrete primers at each position to handle intraspecies diversity.
Primer sequences
https://github.com/quick-lab/primerschemes/blob/main/primerschemes/hbv/600/v2.1.0/primer.bed
The amplicons can also be fragmented and sequenced on Illumina platforms.
Recommended extraction protocol
We use the QIAamp minelute virus spin kit with carrier RNA. For samples with VL >5log IU/ml we extract from 200ul of sample using the manufacturers protocol, for samples with VL <5log IU/ml we extract from 400ul sample doubling up the protease, AL and ethanol.

Materials

A	B	C
**Component**	**Supplier**	**Part number**
HEP-TILE primers hbv/600/v2.1.0	IDT	See links below
Q5 Hot Start High-Fidelity 2X Master Mix	NEB	M0494
Nuclease-free water (100 mL)	NEB	B1500
SPRI-select beads	Beckman	B23318
Ethanol		
NEBNext Ultra II End Repair/dA-tailing module	NEB	E7546
Blunt/TA Ligase Master Mix	NEB	M0367
NEBNext Quick Ligation Module	NEB	E6056S
Native Barcoding Kit 24 V14 or	ONT	SQK-NBD114.24
Native Barcoding Kit 96 V14	ONT	SQK-NBD114.96
Native Barcoding Auxiliary Kit V14 (optional)	ONT	EXP-NBA114
Short Fragment Buffer Expansion Kit	ONT	EXP-SFB001
Flow Cell Priming Kit (optional)	ONT	EXP-FLP004
Flow Cell Wash Kit (optional)	ONT	EXP-WSH004
R10.4.1 flow cells	ONT	FLO-MIN114
Bovine serum albumin (50mg/ml)	Invitrogen	
AMPure XP beads	Beckman	A63881
Qubit dsDNA HS Assay Kit	Thermo	Q32854

Order oligos listed here individually:
https://github.com/quick-lab/primerschemes/tree/main/primerschemes/hbv/600/v2.1.0

Before start

Prepare between 11 and 95 DNA samples plus 1 negative control using this protocol.

Sample preparation

1 Prepare between 11 and 94 DNA samples plus 1 negative control of nuclease-free water per library (a 2nd negative control is added at the start of library prep).
Batch samples by viral load, processing samples above/below 5 log10 IU/ml separately.
If previously frozen, mix by briefly vortexing and pulse spin to collect liquid. Keep samples on ice at all times.

Note

A positive control can also be included which may be a the NIBSC HBV control or clinical sample. This can help monitor run performance.

Primer pool preparation

2 If making up primer pools from individual oligos fully resuspend lyophilised oligos in 1xTE to a concentration of [M] 100 micromolar (µM) , vortex thoroughly and spin down.

3 Sort all odd regions primers into one or more tube racks. Add 5 µL of each odd region primer to a 1.5 mL Eppendorf tube labelled "Pool 1 ([M] 100 micromolar (µM))". Repeat the process for all even region primers for Pool 2. These are your [M] 100 micromolar (µM) stocks of each primer pool.

Note

Primers should be diluted and pooled in the **mastermix** cabinet which should be cleaned with decontamination wipes and UV sterilised before and after use.

Note

For more information see Figure 2 in;

Quick, J. et al. Multiplex PCR method for MinION and Illumina sequencing of Zika and other virus genomes directly from clinical samples. Nat Protoc 12, 1261–1276 (2017). https://doi.org/10.1038/nprot.2017.066

4 Dilute [M] 100 micromolar (µM) pools 1:10 in molecular grade water, to generate [M] 10 micromolar (µM) primer stocks.

Note

Primers are used at a final concentration of [M] 15 nanomolar (nM) per primer. In this case hbv/600/v2.1.0 pools have 69 primers in pool 1 and 63 primers in pool 2. so the requirement is ~ 2.5 µL primer pool ([M] 10 micromolar (µM)) per 25 µL reaction.

Note

Make up multiple 100 µL aliquots of [M] 10 micromolar (µM) primer dilutions and freeze them in case of degradation or contamination.

Multiplex PCR

5 Set up the two PCR reactions per sample as follows in strip-tubes or plates. Gently mix by pipetting and pulse spin the tube to collect liquid at the bottom of the tube.

Samples > 5log10 IU/ml

A	B	C
**Component**	**Reaction 1**	**Reaction 2**
Q5 Hot Start High-Fidelity 2X Master Mix	12.5 µL	12.5 µL
V3 Pool 1 (10µM)	2.5 µL	0 µL
V3 Pool 2 (10µM)	0 µL	2.5 µL
Nuclease-free water	7.5 µL	7.5 µL
(Sample)	(2.5ul)	(2.5ul)
**Total**	**25 µL**	**25 µL**

Samples < 5log10 IU/ml

A	B	C
**Component**	**Reaction 1**	**Reaction 2**
Q5 Hot Start High-Fidelity 2X Master Mix	12.5 µL	12.5 µL
V3 Pool 1 (10µM)	2.5 µL	0 µL
V3 Pool 2 (10µM)	0 µL	2.5 µL
Nuclease-free water	2.5 µL	2.5 µL
(Sample)	(7ul)	(7ul)
**Total**	**25 µL**	**25 µL**

Note

Up to 7 µL DNA can be added to each PCR reaction (in place of nuclease-free water) to improve amplification of low titre samples.
> 5 log10 IU/ml use 2.5ul
< 5 log10 IU/ml use 7ul

PCR reactions can be performed in duplicate for low viral load samples e.g. < 3 log10 IU/ml

Note

To prevent pre-PCR contamination the mastermix for each pool should be made up in the **mastermix** cabinet which should should be cleaned with decontamination wipes and UV sterilised before and after use and aliquoted into PCR strip-tubes/plate

6 Add DNA to each of the PCR reactions (volume determined by VL of sample, see step 5), gently mix by pipetting and pulse spin the tube to collect liquid at the bottom of the tube.

Note

DNA should be added in the **DNA/sample addition** cabinet which should should be cleaned with decontamination wipes and UV sterilised before and after use.

7 Set-up the following program on the thermal cycler, use 35 cycles if VL > 5 log10 IU/ml, use 40 cycles if VL < 5 log10 IU/ml.

**Step**	**Temperature**	**Time**	**Cycles**
Heat Activation	98 °C	00:00:30	1
Denaturation	98 °C	00:00:15	35 or 40
Annealing	65 °C	00:05:00	35 or 40
Hold	4 °C	Indefinite	1

Bead clean up

8 Label strip-tubes/plate and combine the following volumes of each PCR reaction for 25 µL each sample:

A	B
**Component**	**Volume**
Pool 1 PCR reaction	25 µL
Pool 2 PCR reaction	25 µL
Total	50 µL

Note

Amplicons should be added in the **post-PCR** cabinet which should should be cleaned with decontamination wipes and UV sterilised before and after use.

9 Add 40 µL SPRI beads to 50 µL pooled PCR product for an 0.8x clean up. Gently mix and incubate for 00:05:00 at room temperature 5m

9.1 Place on a magnetic rack and incubate for 00:02:00 or until the beads have pelletted and supernatant is completely clear. Remove and discard the supernatant. 2m

9.2 Perform an ethanol wash: add 100 µL of room temperature 70% ethanol to bathe the pellet, carefully remove and discard ethanol being careful not to touch the bead pellet.

9.3 Repeat the ethanol wash (step 9.2)

9.4 Pulse centrifuge to collect and remove residual ethanol, remove excess with a P10 pipette, it is very important all is removed.

9.5 With the tube lid open, air dry for 1-2 minutes, or until the pellet loses its shine. Do not over-dry.

9.6 Re-suspend pellet in 20 µL H20, mix gently and incubate for 2 minutes at room temperature

9.7 Place on magetic rack until clear, transfer the supernatant to clean labelled tubes, do not disturb the beads.

9.8 Quantify using a Qubit or Quantus.

End preparation

10 In a new PCR strip-tube/plate set up the following reaction for each sample, normalising to 80ng amplicon DNA input per sample. Add an additional reagent-only negative control at this step.

10.1

A	B
**Component**	**Volume**

A	B
Clean PCR product from previous step	Up to 8.3 ul
Ultra II end prep reaction buffer	1.2ul
Ultra II end prep enzyme mix	0.5ul
Nuclease-free water	to a total of 10ul
**Total**	**10ul**

Note

Make a master mix of end-preparation reagents and nuclease-free water and aliquot into strip-tube/plate to improve reproducability.

10.2 Incubate at room temperature for 00:15:00 31m

Incubate at 65 °C for 00:15:00

Incubate on ice for 00:01:00

Native barcoding

11 In a new PCR strip-tube/plate set up the following reaction for each sample:

11.1

A	B
**Component**	**Volume**
End-preparation reaction mixture	1 ul
NBXX barcode	1.25 ul
Blunt/TA Ligase master mix	5 ul
Nuclease-free water	2.75 ul
**Total**	**10 ul**

Note

Use one native barcode per sample. Use 12 or more barcodes per library or there will be insufficient total material to achieve good yields.

11.2 Incubate at room temperature for 00:20:00 20m

11.3 Add EDTA and mix thoroughly

A	B
**EDTA cap colour**	**Volume per well**
For clear cap EDTA	1ul
For blue cap EDTA	2ul

Note

NB. EDTA concentration varies by cap colour supplied by ONT

Note

Alternatively a 65C heat step can be used to inactivate the ligase - see ONT version of protocol.

11.4 In a new 1.5ml Eppendorf tube pull all one-pot barcoding reactions together.

Note

If processing 12-24 samples pool all 10 µL from each native barcoding reaction.
if processing 48 samples pool 5 µL from each native barcoding reaction.
If processing 96 samples pool 2.5 µL from each native barcoding reaction so as not to exceed a pool volume of 240 µL which would make the clean-up volume too large.

11.5 Determine volume of pooled barcoded samples, add an equal volume of water

11.6 Add ampure beads (supplied with ONT kit) for a 1.5x clean up. If very large volume split between 2 tubes
Example:

A	B
**Component**	**Example volumes**
Pooled barcoded samples	240 ul
Water	240 ul
Beads	720 ul

Note

NB. 0.4x clean up in ONT protocol was leading to loss of whole library.

11.7 Mix by vortexing and pulse centrifuge to collect all liquid at the bottom of the tube. Incubate for 00:05:00 at room temperature. 5m

11.8 Place on magnetic rack and incubate for 00:02:00 or until the beads have pelleted and the supernatant is completely clear. Carefully remove and discard the supernatant, being careful not to touch the bead pellet. 2m

11.9 Add 250 µL SFB and resuspend beads completely by pipette mixing. Pulse centrifuge to collect all liquid at the bottom of the tube and place on the magnet. Remove supernatant and discard.

11.10 Repeat step 11.9 to perform a second SFB wash. Pulse centrifuge and remove any residual SFB.

Note

You do not need to allow to air dry with SFB washes.

11.11 Add 200 µL of room-temperature 70 % ethanol to bathe the pellet. Carefully remove and discard ethanol, being careful not to touch the bead pellet.

Note

Only perform 1x 70% ethanol wash

11.12 Pulse centrifuge to collect all liquid at the bottom of the tube and carefully remove as much residual ethanol as possible using a P10 pipette.

11.13 With the tube lid open incubate for 00:03:00 or until the pellet loses it's shine (very large pellets will take longer, NB if the pellet dries completely it will crack and become difficult to resuspend). 3m

11.14 00:02:00 Re-suspend pellet in 31 µL nuclease free water, mix gently by either flicking or pipetting and incubate for 00:02:00 . If beads were split between two eppendorfs then combine at this point to elute in a total of 31ul. 4m

11.15 Place on magnet and transfer sample to a clean 1.5 mL Eppendorf tube ensuring no beads are transferred into this tube.

12 Quantify 1 µL of the barcoded amplicons using a fluorometer such as a Qubit or Quantus. Concentration will vary depending on number and Ct of samples and but you need about

30 ng total at this stage to achieve maximum run yield.

Adaptor ligation

13 Set up the following Native Adapter (NA) ligation and clean-up with SFB.

14 In a new 1.5ml eppendorf or PCR tube set up the following adapter ligation reaction

A	B
**Component**	**Volume**
Barcoded amplicon pool	30 ul
NEBNext quick ligation reaction buffer (5X)	10 ul
Adaptor mix (NA)	5 ul
Quick T4 DNA ligase	5 ul
**Total**	**50 ul**

14.1 Incubate at room temperature for 00:20:00 20m

15 Perform a bead clean up.

15.1 Add 50ul ampure beads (supplied with ONT kit) to the sample tube. Mix by vortexing and pulse centrifuge to collect all liquid at the bottom of the tube. Incubate for 00:05:00 at room temperature.

15.2 Place on magnetic rack and incubate for 00:02:00 or until the beads have pelleted and the supernatant is completely clear. Carefully remove and discard the supernatant, being careful not to touch the bead pellet.

15.3 Add 250 µL SFB and resuspend beads completely by pipette mixing. Pulse centrifuge to collect all liquid at the bottom of the tube and place on the magnet. Remove supernatant and discard.

15.4 Repeat step 15.3 to perform a second SFB wash. Pulse centrifuge and remove any residual SFB.

Note

You do not need to allow to air dry with SFB washes.

15.5 Re-suspend pellet in 15 µL EB (ONT), mix gently by either flicking or pipetting and incubate for 00:02:00 2m

15.6 Place on magnet and transfer sample to a clean 1.5 mL Eppendorf tube ensuring no beads are transferred into this tube.

16 Quantify the final library using a fluorometer such as a Qubit or Quatus.

Note

Concentration will vary depending on number and Ct of samples, expected quantification between 2-9 ng/ul, 15 ng final library is usually required to acheive maximum run yield.

Note

Final library can be now be stored at 4 °C for up to a week if needed otherwise proceed directly to MinION sequencing.

Flow cell check and priming

17 Refer to ONT documentation for images explaining how to check, prime and load flow cell.

18 Complete a flow cell check - open Minknow user interface, plug in MinION to laptop, open the MinION lid and slide the flow cell under the clip. Press down firmly on the flow cell to ensure correct thermal and electrical contact. Click flow cell check on Minknow interface.

19 To prepare the flow cell priming mix, combine the following reagents in the FCF tube and mix by inverting the tube and pipette mix at room temperature:

A	B
**Component**	**Volume**
Flow cell flush (FCF)	1170 ul
Bovine serum albumin (BSA) at 50mg/ml	5 ul
Flow cell tether (FCT)	30 ul
**Final volume in FCF tube**	**1205 ul**

19.1 Slide the flow cell priming port cover clockwise to open the priming port

19.2 After opening the priming port, set a P1000 pipette to 200ul, insert the tip into the priming port, turn the wheel until the dial shows 220-230ul, to draw back 20-30ul or until you can see a small volume of buffer entering the pipette tip.

19.3 Visually check that there is continuous buffer from the priming port across the sensor array.

19.4 Load 800 µL of the priming mix into the flow cell via the priming port, avoiding the introduction of air bubbles. Wait for five minutes. During this time prepare the library for loading by following the steps below.

MinION sequencing

20 Thoroughly mix the contents of the library beads by pipetting, it is vial that they are mixed immediately before use.

Note

From experience we have successfully loaded 20ng library

21 In a new 1.5ml Eppendorf DNA LoBind tube, prepare the library for loading as follows (NB load the library onto flow cell immediately after adding SB and LIB because the fuel in the buffer will start to be consumed by the adapter):

A	B
**Reagent**	**Volume**
Sequencing buffer (SB)	37.5 ul
Library beads (LIB) mixed immediately before use	25.5 ul
DNA library + H2O	12 ul
**Total**	**75 ul**

22 Complete flow cell priming: lift the spot on sample port cover, load 200 µL of the priming mix into the flow cell priming port (not the SpotON sample port), avoiding the introduction of air bubbles

23 Mix the prepared library gently by pipetting just prior to loading

24 Add half of the library (~37.5ul) to the flow cell via the SpotON sample port in a dropwise fashion. Ensure each drop flows into the port before adding the next. Resuspend beads/library and add the 2nd half in a dropwise fashion.

25 Gently replace the SpotON sample port cover, making sure the bung enters the SpotON port and close the priming port.

26 Place the light shield onto the flow cell. And close the device lid.

27 Start the sequencing run using MinKnow.

Note

If using live basecalling, turn on double-ended barcoding in the basecalling settings.

Protocol references

Pre-print coming soon: Whole genome sequencing of hepatitis B virus (HBV) using tiled amplicon (HEP-TILE) and probe-based enrichment on Illumina and Nanopore platforms. Lumley et al. 2024
