# Supplementary material for: Whole genome sequencing of hepatitis B virus using tiled amplicon (HEPTILE) and probe based enrichment on Illumina and Nanopore platforms
Source: Sci Rep. 2025 Feb 17;15:5795. doi: 10.1038/s41598-025-87721-1 (PMC11832747; doi:10.1038/s41598-025-87721-1)
Supplement: Supplementary file 4 — Supplementary Material 4 [file 41598_2025_87721_MOESM4_ESM.docx]

**Supplementary materials: Lumley et al. Whole genome sequencing of hepatitis B virus using tiled amplicon (HEPTILE) and probe based enrichment on Illumina and Nanopore platforms**

**Supplementary material**

**Supplementary figures**

Supplementary figure 1: Modifications to primer positions to take into account HBV genome structure.

Supplementary figure 2: Relationship between HBV genotype and performance of HEP-TILE Nanopore workflow.

**Supplementary tables**

Supplementary table 1: Sequencing metrics for mock HBV samples for capture-Illumina, HEP-TILE Nanopore and HEP-TILE Illumina workflows vs. no enrichment

Supplementary table 2: Metadata and sequencing outputs for clinical HBV samples sequenced with capture-Illumina, HEP-TILE Nanopore and HEP-TILE Illumina workflows.

Supplementary table 3: Number of sequences downloaded from publicly available sequence database HBVdb

Supplementary table 4: HBV probe sequences

Supplementary table 5: initial 8 amplicon scheme (hbv/500/v1.1.0)

Supplementary table 6: final 6 amplicon scheme (hbv/600/v2.1.0/)

**Supplementary text**


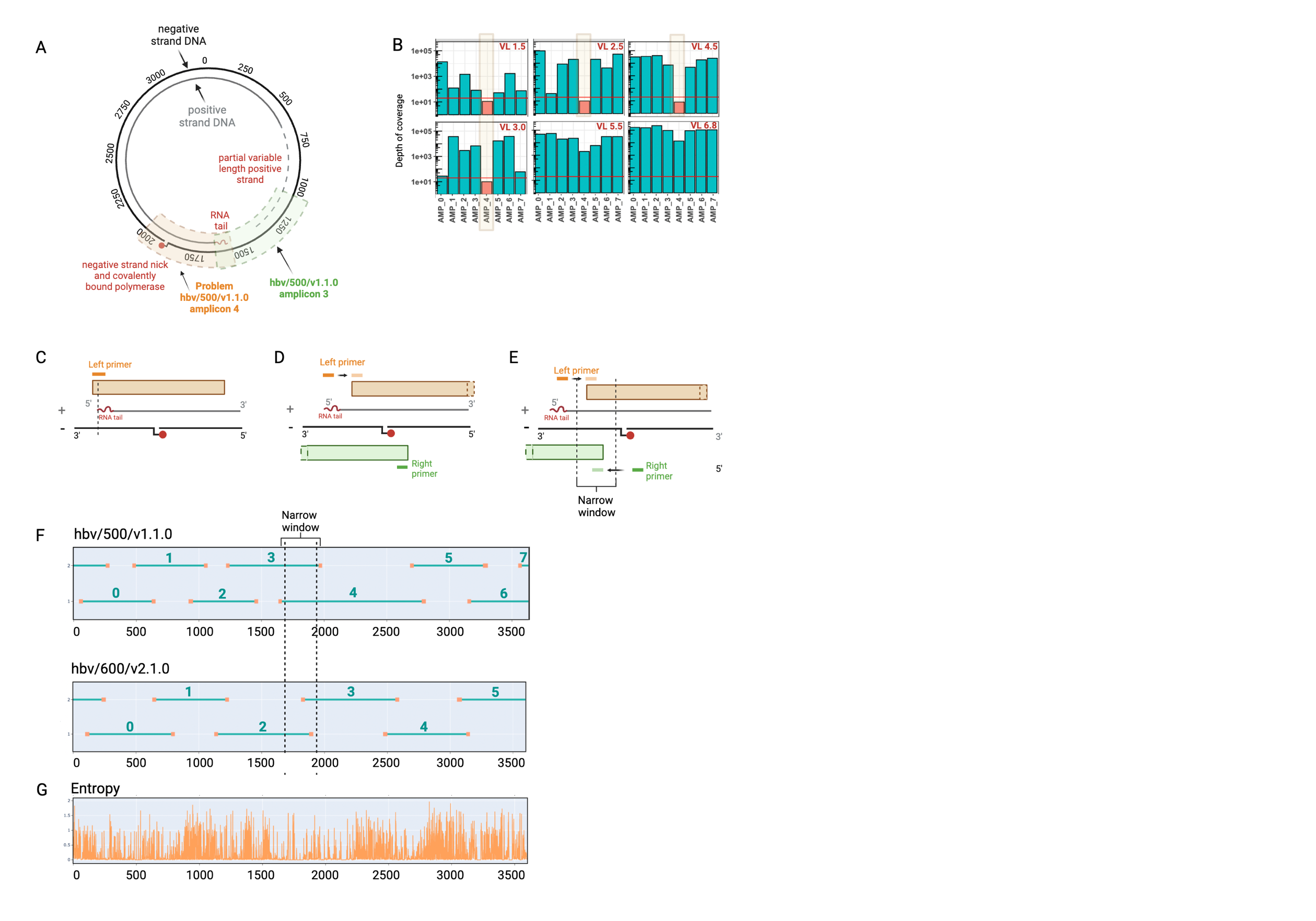


**Supplementary figure 1: Modifications to primer positions to take into account HBV genome structure.** (A) Schematic of HBV relaxed circular DNA (rc-DNA), the HBV partially dsDNA genome has 5’ modifications to both the positive DNA strand (RNA tail) and negative DNA strand (covalently bound polymerase). The negative strand is complete but not fully circular due to the presence of a ‘nick’ and covalently bound polymerase. The partial positive strand is variable in length. (B) Depth of coverage for amplicon scheme hbv/500/v1.1.0 - illustrating drop-out of hbv/500/v1.1.0 amplicon 4 in samples below a VL of 5 log_10_ IU/ml (VL shown in red text). (C) hbv/500/v1.1.0 amplicon 4 drops out in low-medium viral load samples, the hypothesis is that neither the positive nor negative strands of the HBV relaxed circular genome are complete in that region. The left hand primer only partially overlaps the 5’ tail of the positive strand, the negative strand contains a nick. (D) Attempt to solve issue by moving the hbv/500/v1.1.0 amplicon 4 primer upstream, however this has the knock on effect of the Amplicon 3 right hand primer moving upstream, which now means amplicon 3 falls across a non-continuous DNA region. (E) Solution requires the left hand primer for amplicon 4 and right hand primer for amplicon 3 to be placed in a narrow 200bp window, to ensure green can be generated from the positive DNA strand, and orange from the negative DNA strand. (F) Locations and numbering of primers for initial 8 amplicon primer scheme (hbv/500/v1.1.0) and updated 5 amplicon primer scheme (hbv/600/v2.1.0) (G) Entropy across HBV genome. Created in BioRender. Lumley, S. (2024) BioRender.com/l10u171


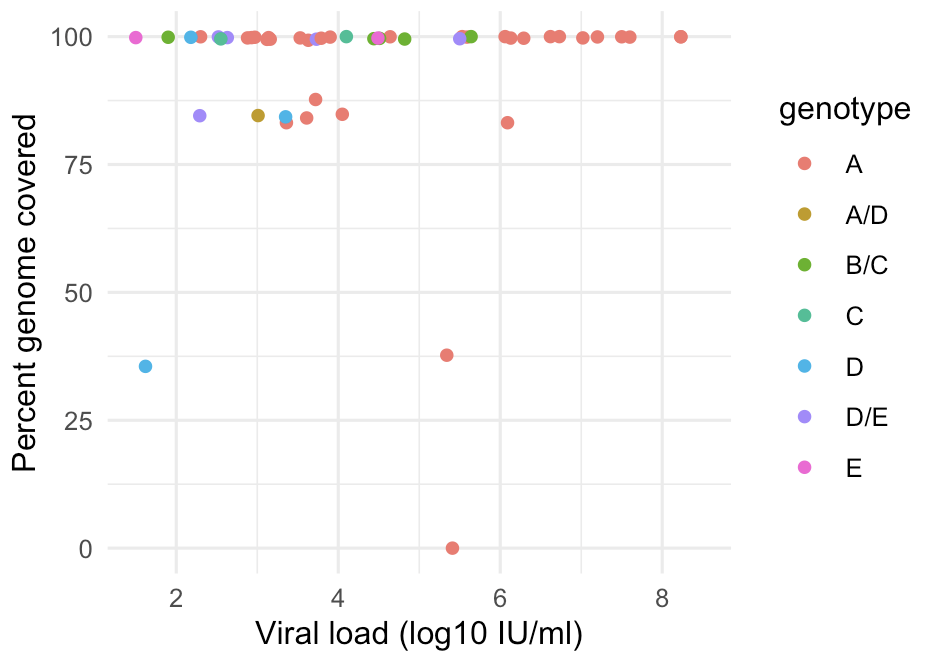


**Supplementary figure 2: Relationship between HBV genotype and performance of HEP-TILE Nanopore workflow.** Percentage genome coverage shown at x20 depth.


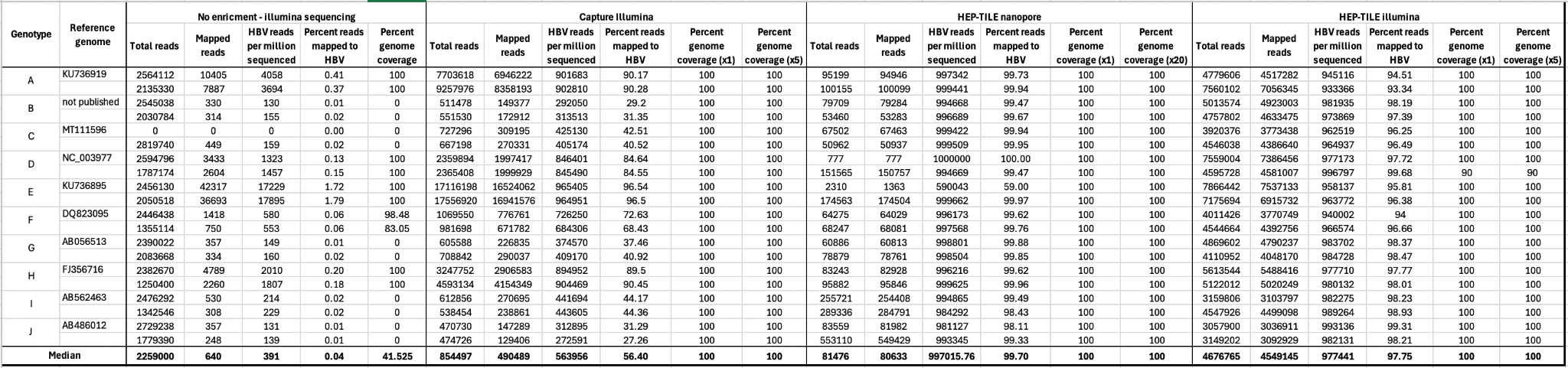


**Supplementary table 1: Sequencing metrics for mock HBV samples for capture-Illumina, HEP-TILE Nanopore and HEP-TILE Illumina workflows vs. no enrichment**

**
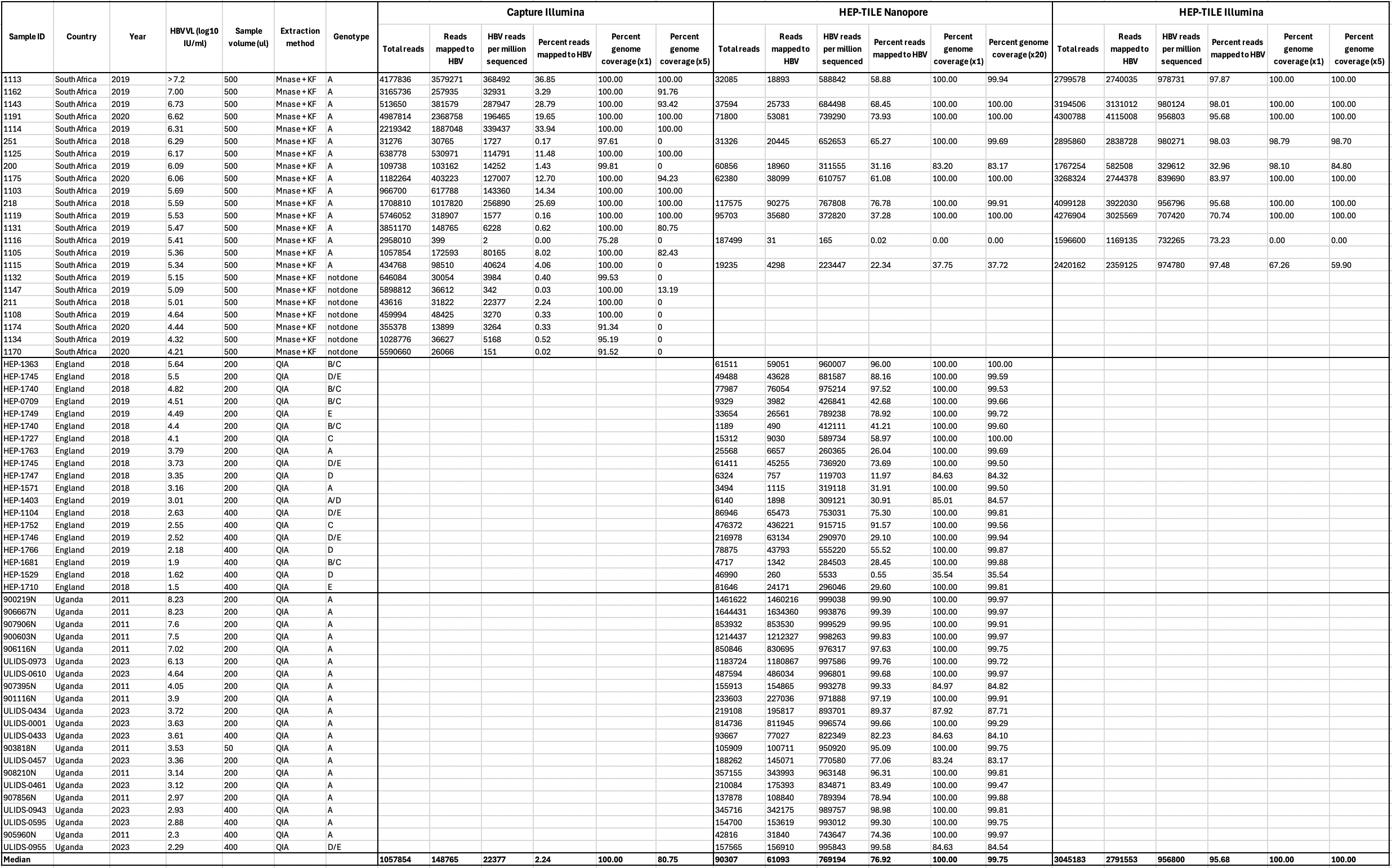
**

**Supplementary table 2: Metadata and sequencing outputs for clinical HBV samples sequenced with capture-Illumina, HEP-TILE Nanopore and HEP-TILE Illumina workflows.** Sequencing metrics for sequencing of HBV-positive clinical samples.

Key for extraction methods (see methods section for full details): “Mnase + KF” = host depletion with micrococcal nuclease followed by extraction on Kingfisher apex, “QIA” = QiaAMP MinElute virus spin kit. Viral load conversion example: 2log_10_ IU/ml = 100 IU/ml, 3log_10_ IU/ml = 1000 IU/ml.

| **Genotype** | **Capture**  **(2019 HBVdb download)** | | **HEP-TILE**  **(2024 HBVdb download)** | |
| --- | --- | --- | --- | --- |
|  | **Genomes in HBVdb** | **Genomes used** | **Genomes in HBVdb** | **Downsampled Genomes** |
| **A** | 506 | 506 | 1066 | 559 |
| **B** | 1218 | 1218 | 2018 | 1143 |
| **C** | 1447 | 1447 | 3095 | 1764 |
| **D** | 823 | 823 | 1523 | 920 |
| **E** | 254 | 254 | 397 | 254 |
| **F** | 197 | 197 | 304 | 156 |
| **G** | 28 | 28 | 50 | 18 |
| **H** | 26 | 26 | 28 | 17 |

**Supplementary table 3: Number of sequences downloaded from publicly available sequence database HBVdb**[20]

**Supplementary table 4: HBV probe sequences**

>probe_1

CTCAACACAGTTCCACCAAGCTCTGCAAGATCCGAGAGTAAGGGGCCTGTATTTTCCTGCTGGTGGCTCCAGTTCAGAAACAGAGAACCCTGTTCCGACTATTGCCTCTCTCACATCATC

>probe_2

TGGTGGCTCCAGTTCAGAAACAGAGAACCCTGTTCCGACTATTGCCTCTCTCACATCATCAATCTTCTCGAAGACTGGGGACCCTGCAATGAACATGGAGAACATCACATCAGGACTCCT

>probe_3

AATCTTCTCGAAGACTGGGGACCCTGCAATGAACATGGAGAACATCACATCAGGACTCCTAGGACCCCTGCTCGTGTTACAGGCGGGGTGTTTCTTGTTGACAAAAATCCTCACAATACC

>probe_4

AGGACCCCTGCTCGTGTTACAGGCGGGGTGTTTCTTGTTGACAAAAATCCTCACAATACCGCAGAGTCTAGACTCGTGGTGGACTTCTCTCAATTTTCTAGGGGGACCACCCGGGTGTCC

>probe_5

GCAGAGTCTAGACTCGTGGTGGACTTCTCTCAATTTTCTAGGGGGACCACCCGGGTGTCCTGGCCAAAATTCGCAGTCCCCAACCTCCAATCACTCACCAACCTCCTGTCCTCCAACTTG

>probe_6

TGGCCAAAATTCGCAGTCCCCAACCTCCAATCACTCACCAACCTCCTGTCCTCCAACTTGTCCTGGCTATCGCTGGATGTGTCTGCGGCGTTTTATCATCTTCCTCTTCATCCTGCTGCT

>probe_7

TCCTGGCTATCGCTGGATGTGTCTGCGGCGTTTTATCATCTTCCTCTTCATCCTGCTGCTATGCCTCATCTTCTTGTTGGTTCTTCTGGACTATCAAGGTATGTTGCCCGTTTGTCCTCT

>probe_8

ATGCCTCATCTTCTTGTTGGTTCTTCTGGACTATCAAGGTATGTTGCCCGTTTGTCCTCTAATTCCAGGATCCACAACCACCAGCACGGGACCCTGCAAAACCTGCACGACTCCTGCTCA

>probe_9

AATTCCAGGATCCACAACCACCAGCACGGGACCCTGCAAAACCTGCACGACTCCTGCTCAAGGAACCTCTATGTTTCCCTCATGTTGCTGTACAAAACCTTCGGACGGAAATTGCACCTG

>probe_10

AGGAACCTCTATGTTTCCCTCATGTTGCTGTACAAAACCTTCGGACGGAAATTGCACCTGTATTCCCATCCCATCATCTTGGGCTTTCGGAAAATACCTATGGGAGTGGGCCTCAGCCCG

>probe_11

TATTCCCATCCCATCATCTTGGGCTTTCGGAAAATACCTATGGGAGTGGGCCTCAGCCCGTTTCTCCTGGCTCAGTTTACTAGTGCAATTTGTTCAGTGGTGCGTAGGGCTTTCCCCCAC

>probe_12

TTTCTCCTGGCTCAGTTTACTAGTGCAATTTGTTCAGTGGTGCGTAGGGCTTTCCCCCACTGTCTGGCTTTCAGTTATATGGATGATGTGGTATTGGGGGCCAAATCTGTGCAGCATCTT

>probe_13

TGTCTGGCTTTCAGTTATATGGATGATGTGGTATTGGGGGCCAAATCTGTGCAGCATCTTGAGTCCCTTTATACCGCTGTTACCAATTTTCTGTTGTCTGTGGGTATACATTTAAACACT

>probe_14

GAGTCCCTTTATACCGCTGTTACCAATTTTCTGTTGTCTGTGGGTATACATTTAAACACTAACAAAACAAAAAGATGGGGTTATTCCCTAAATTTCATGGGTTATGTAATTGGAAGTTGG

>probe_15

AACAAAACAAAAAGATGGGGTTATTCCCTAAATTTCATGGGTTATGTAATTGGAAGTTGGGGGACATTGCCACAAGAACATATTGTACAAAAAATCAAAGAATGTTTTCGAAAACTTCCT

>probe_16

GGGACATTGCCACAAGAACATATTGTACAAAAAATCAAAGAATGTTTTCGAAAACTTCCTGTTAATAGGCCTATTGATTGGAAAGTCTGTCAACGAATTGTGGGTCTTTTGGGCTTTGCT

>probe_17

GTTAATAGGCCTATTGATTGGAAAGTCTGTCAACGAATTGTGGGTCTTTTGGGCTTTGCTGCCCCTTTTACCCAATGTGGTTATCCTGCTCTAATGCCTTTGTATGCATGTATTAAAGCT

>probe_18

GCCCCTTTTACCCAATGTGGTTATCCTGCTCTAATGCCTTTGTATGCATGTATTAAAGCTAAGCAGGCTTTTGCTTTCTCGCCAACTTACAAGGCCTTTCTCTGTAAACAATACATGAAC

>probe_19

AAGCAGGCTTTTGCTTTCTCGCCAACTTACAAGGCCTTTCTCTGTAAACAATACATGAACCTTTACCCCGTTGCTCGGCAACGGCCAGGCCTGTGCCAAGTGTTTGCTGACGCAACCCCC

>probe_20

CTTTACCCCGTTGCTCGGCAACGGCCAGGCCTGTGCCAAGTGTTTGCTGACGCAACCCCCACTGGCTGGGGCTTGGCCATTGGCCATCAGCGCATGCGTGGAACCTTTGTGGCTCCTCTG

>probe_21

ACTGGCTGGGGCTTGGCCATTGGCCATCAGCGCATGCGTGGAACCTTTGTGGCTCCTCTGCCGATCCATACTGCGGAACTCCTAGCAGCTTGTTTCGCTCGCAGCCGGTCTGGAGCGAAA

>probe_22

CCGATCCATACTGCGGAACTCCTAGCAGCTTGTTTCGCTCGCAGCCGGTCTGGAGCGAAACTTATCGGGACTGACAACTCTGTTGTCCTCTCTCGGAAGTACACCTCCTTTCCATGGCTG

>probe_23

CTTATCGGGACTGACAACTCTGTTGTCCTCTCTCGGAAGTACACCTCCTTTCCATGGCTGCTAGGCTGTGCTGCCAACTGGATCCTGCGCGGGACGTCCTTTGTTTACGTCCCGTCGGCG

>probe_24

CTAGGCTGTGCTGCCAACTGGATCCTGCGCGGGACGTCCTTTGTTTACGTCCCGTCGGCGCTGAATCCCGCGGACGACCCCTCTCGGGGTCGCTTGGGGCTCTATCGCCCCCTTCTCCGT

>probe_25

CTGAATCCCGCGGACGACCCCTCTCGGGGTCGCTTGGGGCTCTATCGCCCCCTTCTCCGTCTGCCGTTCCGGCCGACGACGGGGCGCACCTCTCTTTACGCGGACTCCCCGTCTGTGCCT

>probe_26

CTGCCGTTCCGGCCGACGACGGGGCGCACCTCTCTTTACGCGGACTCCCCGTCTGTGCCTTCTCATCTGCCGGACCGTGTGCACTTCGCTTCACCTCTGCACGTCGCATGGAGACCACCG

>probe_27

TCTCATCTGCCGGACCGTGTGCACTTCGCTTCACCTCTGCACGTCGCATGGAGACCACCGTGAACGCCCCTCAGAGCTTGCCAAAGGTCTTACATAAGAGGACTCTTGGACTTTCAGCAA

>probe_28

TGAACGCCCCTCAGAGCTTGCCAAAGGTCTTACATAAGAGGACTCTTGGACTTTCAGCAAGGTCAACGACCGGGATTGAGGAATACATCAAAGACTGTGTATTTAAGGACTGGGAGGAGT

>probe_29

GGTCAACGACCGGGATTGAGGAATACATCAAAGACTGTGTATTTAAGGACTGGGAGGAGTTGGGGGAGGAGATTAGGTTAAAGGTCTTTGTATTAGGAGGCTGTAGGCATAAATTGGTCT

>probe_30

TGGGGGAGGAGATTAGGTTAAAGGTCTTTGTATTAGGAGGCTGTAGGCATAAATTGGTCTGTGCACCAGCACCATGCAACTTTTTCACCTCTGCCTAATCATCTCTTGTTCATGTCCTAC

>probe_31

GTGCACCAGCACCATGCAACTTTTTCACCTCTGCCTAATCATCTCTTGTTCATGTCCTACTGTTCAAGCCTCCAAGCTGTGCCTTGGGTGGCTTTGGGGCATGGATAGAACAACTTTGCC

>probe_32

TGTTCAAGCCTCCAAGCTGTGCCTTGGGTGGCTTTGGGGCATGGATAGAACAACTTTGCCATATGGCCTTTTTGGCTTAGACATTGACCCTTATAAAGAATTTGGAGCTTCTGTGGAGTT

>probe_33

ATATGGCCTTTTTGGCTTAGACATTGACCCTTATAAAGAATTTGGAGCTTCTGTGGAGTTACTCTCGTTTTTGCCTTCTGACTTCTTCCCGTCTGTTCGGGACCTACTCGACACCGCTTC

>probe_34

ACTCTCGTTTTTGCCTTCTGACTTCTTCCCGTCTGTTCGGGACCTACTCGACACCGCTTCAGCCCTGTACCGGGATGCCTTAGAGTCACCTGAACATTGCACACCTAACCATACAGCACT

>probe_35

AGCCCTGTACCGGGATGCCTTAGAGTCACCTGAACATTGCACACCTAACCATACAGCACTCAGGCAAGCTATTCTGTGCTGGGGTGAGTTAATGACTCTGGCTACCTGGGTGGGCAATAA

>probe_36

CAGGCAAGCTATTCTGTGCTGGGGTGAGTTAATGACTCTGGCTACCTGGGTGGGCAATAATTTGGAAGATCCAGCAGCCAGGGATTTAGTAGTCAATTATGTCAATACTAATATGGGCCT

>probe_37

TTTGGAAGATCCAGCAGCCAGGGATTTAGTAGTCAATTATGTCAATACTAATATGGGCCTAAAAATCAGACAACTATTGTGGTTTCACATTTCCTGCCTTACTTTTGGAAGAGAAACTGT

>probe_38

AAAAATCAGACAACTATTGTGGTTTCACATTTCCTGCCTTACTTTTGGAAGAGAAACTGTTCTTGAGTATTTGGTGTCTTTTGGAGTGTGGATTCGCACTCCTCCTGCTTATAGACCACC

>probe_39

TCTTGAGTATTTGGTGTCTTTTGGAGTGTGGATTCGCACTCCTCCTGCTTATAGACCACCAAATGCCCCTATCCTATCAACACTTCCGGAAACTACTGTTGTTAGACGACGGGACCGAGG

>probe_40

AAATGCCCCTATCCTATCAACACTTCCGGAAACTACTGTTGTTAGACGACGGGACCGAGGCAGGTCCCCTAGAAGAAGAACTCCCTCGCCTCGCAGACGAAGATCTCAATCGCCGCGTCG

>probe_41

CAGGTCCCCTAGAAGAAGAACTCCCTCGCCTCGCAGACGAAGATCTCAATCGCCGCGTCGCAGAAGATCTCAATCTCCAGCTTCCCAATGTTAGTATTCCTTGGACTCATAAGGTGGGAA

>probe_42

CAGAAGATCTCAATCTCCAGCTTCCCAATGTTAGTATTCCTTGGACTCATAAGGTGGGAAACTTTACGGGGCTTTATTCTTCTACTGTACCTGTCTTTAATCCTGACTGGCAAACTCCTT

>probe_43

ACTTTACGGGGCTTTATTCTTCTACTGTACCTGTCTTTAATCCTGACTGGCAAACTCCTTCTTTTCCTGATATTCATTTGCATCAAGACATGATAAATAAATGTGAACAATTTGTAGGCC

>probe_44

CTTTTCCTGATATTCATTTGCATCAAGACATGATAAATAAATGTGAACAATTTGTAGGCCCTCTCACAGAAAATGAAAAGAGAAGATTAAAATTAATTATGCCAGCTAGATTTTATCCTA

>probe_45

CTCTCACAGAAAATGAAAAGAGAAGATTAAAATTAATTATGCCAGCTAGATTTTATCCTAACGTTACCAAATATTTGCCTTTGGATAAAGGTATTAAACCTTATTATCCAGAGAATGTAG

>probe_46

ACGTTACCAAATATTTGCCTTTGGATAAAGGTATTAAACCTTATTATCCAGAGAATGTAGTTAATCATTACTTCAAAACCAGACATTATTTACATACTCTATGGAAGGCGGGAATTCTAT

>probe_47

TTAATCATTACTTCAAAACCAGACATTATTTACATACTCTATGGAAGGCGGGAATTCTATATAAGAGAGAAACCACACGTAGCGCCTCATTTTGTGGGTCACCATATTCTTGGGAACAAG

>probe_48

ATAAGAGAGAAACCACACGTAGCGCCTCATTTTGTGGGTCACCATATTCTTGGGAACAAGAGCTACAGCATGGGAGCACCTCGGTCAACGCCTCGAAGGGGCATGGGAAAGAATCTTTCT

>probe_49

AGCTACAGCATGGGAGCACCTCGGTCAACGCCTCGAAGGGGCATGGGAAAGAATCTTTCTGTGCCCAATCCTCTGGGATTCTTTCCAGACCACCAGTTGGATCCAGCATTCAGAGCAAAT

>probe_50

GTGCCCAATCCTCTGGGATTCTTTCCAGACCACCAGTTGGATCCAGCATTCAGAGCAAATTCCAGCAGTCCAGATTGGGACTTCAACACAAACAAGGACAATTGGCCAGAGGCAAACAAG

>probe_51

TCCAGCAGTCCAGATTGGGACTTCAACACAAACAAGGACAATTGGCCAGAGGCAAACAAGGTAGGAGTGGGAGGCTTCGGTCCAGGGTTCACACCCCCACACGGAGGCCTTCTGGGGTGG

>probe_52

GTAGGAGTGGGAGGCTTCGGTCCAGGGTTCACACCCCCACACGGAGGCCTTCTGGGGTGGAGCCCTCAGGCACAGGGCATACTAACAACCTTGCCAGCAGATCCGCCTCCTGCCTCCACC

>probe_53

AGCCCTCAGGCACAGGGCATACTAACAACCTTGCCAGCAGATCCGCCTCCTGCCTCCACCAATCGGCGGTCAGGAAGGAAGCCAACCCCAGTCTCTCCACCTCTAAGAGACACTCATCCA

>probe_54

AATCGGCGGTCAGGAAGGAAGCCAACCCCAGTCTCTCCACCTCTAAGAGACACTCATCCACAGGCCATGCAGTGGAACTCAACACAGTTCCACCAAGCTCTGCAAGATCCGAGAGTAAGG

>probe_55

CAGGCCATGCAGTGGAACTCAACACAGTTCCACCAAGCTCTGCAAGATCCGAGAGTAAGGGGCCTGTATTTTCCTGCTGGTGGCTCCAGTTCAGAAACAGAGAACCCTGTTCCGACTATT

>Deletion_region1903_1939_removed probe

TCTTGTTCATGTCCTACTGTTCAAGCCTCCAAGCTGTGCCTTGGGTGGCTTTGGGGCATGGACATTGACCCTTATAAAGAATTTGGAGCTTCTGTGGAGTTACTCTCGTTTTTGCCTTCT

>Deletion_Genotype_D_region2897_2929_removed probe

ACGTAGCGCATCATTTTGCGGGTCACCATATTCTTGGGAACAAGAGCTACAGCATGGGAGCGAATCTTTCTGTTCCCAACCCTCTGGGATTCTTTCCCGATCATCAGTTGGACCCTGCAT

>H_cons_seq_s2035_e2154_ConS2035

CGAACATTGCACCCCCAACCACACTGCTCTCAGGCAAGCTATTTTGTGCTGGGGTGAGTTGATGACCTTGGCTTCCTGGGTGGGCAATAATTTAGAGGATCCTGCAGCAAGAGATCTAGT

>G_cons_seq_s2845_e2964_ConS2845

TTTGTGGGTCACCATATACTTGGGAACAAGATCTACAGCATGGGGCTTTCTTGGACGGTCCCTCTCGAGTGGGGAAAGAACCTTTCCACCAGCAATCCTCTAGGATTCCTTCCCGATCAC

>G_cons_seq_s1651_e1770_ConS1651

TATATAAGAGGACTCTTGGACTGTTTGTTATGTCAACAACCGGGGTGGAGAAATACTTCAAGGACTGTGTTTTTGCTGAGTGGGAAGAATTAGGCAATGAGTCCAGGTTAATGACCTTTG

>E_cons_seq_s1975_e2094_ConS1975

TTCAGTAAGAGATCTTCTAGATACCGCCTCAGCTCTGTATCGGGATGCCTTAGAATCTCCTGAGCATTGTTCACCTCACCACACTGCACTCAGGCAAGCCATTCTTTGCTGGGGGGAACT

>A_cons_seq_s391_e510_Target_seq391

TTTTATCATATTCCTCTTCATCCTGCTGCTATGCCTTAGCTCTGTATCGGTTCTTCTGAAGCCTTAGAGTCTCCAGAGCATTGCTCCCCTCACCTCAGGATCCACAACAACCAGTACGGG

>A_cons_seq_s2875_e2994_ConS2875

CCTCGCAAAGGCATGGGGACGAATCTTTCTGTTCCCAACCCTCTGGGATTCTTTCCCGATCATCAGTTGGACCCTGCATTCGGAGCCAACTCAAACAATCCAGATTGGGACTTCAACCCC

>Genotype_D_deletion_at_33_55

CACTCATCCTCAGGCCATGCAGTGGAACTCCACAACCTTCCACCAAACTCTGCAAGATCCCCCTGCTGGTGGCTCCAGTTCAGGAACAGTAAACCCTGTTCCGACTACTGTCTCTCCCAT

>D_cons_seq_s2956_e3075_ConS2956

AACAAGGACACCTGGCCAGACGCCAACAAGGTAGGAGCTGGAGCATTCGGGCTGGGATTCACCCCACCGCACGGAGGCCTTTTGGGGTGGAGCCCTCAGGCTCAGGGCATACTACAAACC

>B_cons_seq_s2095_e2214_ConS2095

AATGAATCTAGCCACCTGGGTGGGAAGTAATTTGGAAGATCCAGCATCCAGGGAATTAGTAGTCAGCTATGTCAACGTTAATATGGGCCTAAAAATCAGACAACTATTGTGGTTTCACAT

>B_cons_seq_s1711_e1830_Target_seq1711

AAGACTGTTTGTTTAAAGACTGGGAGGAGTTGGGTTTACTGATTGGGGAGTTGGGGGATGTTCTAAGGGGATGGAAGGCTAAATTGGTCTGTTCACCAGCACCATGCAACTTTTTCACCT

>C_cons_seq_s2990_e3109_ConS2990

ACAAGGATCACTGGCCAGAGGCAAATCAGGTAGGAGCGGGAGCATTCGGGCCAGGGTTCACCCCACCACACGGCGGTCTTTTGGGGTGGAGCCCTCAGGCTCAGGGCATATTGACAACAG

>C_cons_seq_s3080_e3199_ConS3080

GCCCTCAGGCTCAGGGCATATTGACAACAGTGCCAGCAGCACCTCCTCCTGCCTCCACCAATCGGCAGTCAGGAAGACAGCCTACTCCCATCTCTCCACCTCTAAGAGACAGTCATCCTC

>C_cons_seq_s2629_e2748_ConS2629

AATTAATTATGCCTGCTAGGTTCTATCCTAACCTTACCAAATATTTGCCCTTGGACAAAGGCATTAAACCTTATTATCCTGAACATGCAGTTAATCATTACTTCAAAACTAGGCATTATT

>C_cons_seq_s1651_e1770_ConS1651

TACATAAGAGGACTCTTGGACTCTCAGCAATGTCAACGACCGACCTTGAGGCATACTTCAAAGACTGTTTGTTTAAAGACTGGGAGGAGTTGGGGGAGGAGATTAGGTTAATGATCTTTG

>C_cons_seq_s3080_e3199_Target_seq3080

GCCCTCAGGCTCAGGGGACATTGACACCTCCGGCAGCCGCCCCTCCTCCTGCCTCCAGAAAACCTCCTTCAGGTTCTCCACCTACTCTAATTTCTCTTCCTCTAAGAGACAGTCATCCCA

>C_cons_seq_s3140_e3259_ConS3140

AACCTCCTTCAGGTTCTCCACCTACTCTAATTTCTCTTCCTCTAAGAGACAGTCATCCCAACGCCATACACTGCCACTCCACGCTATTCCACCAAGTTCTGCGAGATCCCTTAGTTGCGG

>C_cons_seq_s3200_e3319_ConS3200

AGGCCATGCAGTGGAACTCCACAACATTCCACCAAGCTCTGCTAGATCCCAGAGTGAGGGGCCTATACTTTCCTGCTGGTGGCTCCAGTTCCGGAACAGTAAACCCTGTTCCGACTACTG

**Supplementary table 5: initial 8 amplicon scheme (hbv/500/v1.1.0)**

NB each amplicon has a pool of left and right primers, some of which vary in start/end point by 1-2bp. The positions indicated in the table are a guide, exact locations for all primers are available on the github site. Numbering based on NC_003977.2 reference genome.

| Amplicon  **hbv/500/v1.1.0** | Pool | Strand | Start location | End location | Primer name | Primer sequence |
| --- | --- | --- | --- | --- | --- | --- |
| 0 | 1 | + | 36 | 60 | a1a7f2b2_0_LEFT_0 | AGAGTGAGGGGCCTATACTTTCCT |
|  | 1 | + | 38 | 60 | a1a7f2b2_0_LEFT_1 | AGTCAGGGCCCTGTACTTTCCT |
|  | 1 | + | 35 | 60 | a1a7f2b2_0_LEFT_2 | CAGAGTCAGGGGTCTGTATTTTCCT |
|  | 1 | + | 35 | 60 | a1a7f2b2_0_LEFT_3 | CAGAGTGAGGGGCCTATATTTTCCT |
|  | 1 | + | 34 | 60 | a1a7f2b2_0_LEFT_4 | CCAGAGTAAGAGGCCTGTATTTTCCT |
|  | 1 | + | 34 | 60 | a1a7f2b2_0_LEFT_5 | CCAGAGTAAGGGGCCTATACTTTCCT |
|  | 1 | + | 35 | 60 | a1a7f2b2_0_LEFT_6 | GAGGGTAAGGGCTCTGTATTTTCCT |
|  | 1 | + | 37 | 60 | a1a7f2b2_0_LEFT_7 | GAGTCAGGGCTCTGTACTTTCCT |
|  | 1 | + | 37 | 60 | a1a7f2b2_0_LEFT_8 | GAGTCAGGGGCCTGTATTTTCCT |
|  | 1 | + | 37 | 60 | a1a7f2b2_0_LEFT_9 | GAGTGAGAGGCCTGTATTTCCCT |
|  | 1 | + | 38 | 60 | a1a7f2b2_0_LEFT_10 | GGTGAGAGGCCTGTATTTCCCT |
|  | 1 | - | 584 | 607 | a1a7f2b2_0_RIGHT_0 | GAATACAGGTGCAGTTTCCGTCC |
|  | 1 | - | 584 | 608 | a1a7f2b2_0_RIGHT_1 | GGAATACAAGTGCAGTTTCCGTCC |
|  | 1 | - | 584 | 608 | a1a7f2b2_0_RIGHT_2 | GGAATACAGGTGCAATTTCCGTCC |
|  | 1 | - | 584 | 608 | a1a7f2b2_0_RIGHT_3 | GGAATACAGGTGCAGTTTCCATCC |
|  | 1 | - | 584 | 609 | a1a7f2b2_0_RIGHT_4 | GGGAATACAAGTGCAATTTCCGTCC |
|  | 1 | - | 584 | 609 | a1a7f2b2_0_RIGHT_5 | GGGAATACAGGTGCAATTTCCATCC |
| 1 | 2 | + | 462 | 482 | a1a7f2b2_1_LEFT_0 | ATGTTGCCCGTGTGTCCTCT |
|  | 2 | + | 459 | 482 | a1a7f2b2_1_LEFT_1 | GGTATGTTGCCCGTTTGTCCTCT |
|  | 2 | + | 460 | 482 | a1a7f2b2_1_LEFT_2 | GTATGTTGCCCGTCTGTCCTCT |
|  | 2 | - | 999 | 1022 | a1a7f2b2_1_RIGHT_0 | AGCAAAACCCAAAAGACCCACAA |
|  | 2 | - | 999 | 1023 | a1a7f2b2_1_RIGHT_1 | CAGCAAATCCCAAAAGACCCACAA |
|  | 2 | - | 999 | 1021 | a1a7f2b2_1_RIGHT_2 | GCAAAACCCAGAAGACCCACAA |
|  | 2 | - | 999 | 1021 | a1a7f2b2_1_RIGHT_3 | GCAAACCCCAAAAGACCCACAA |
|  | 2 | - | 999 | 1021 | a1a7f2b2_1_RIGHT_4 | GCAAAGCCCAAAAGACCCACAA |
|  | 2 | - | 999 | 1021 | a1a7f2b2_1_RIGHT_5 | GCAAAGCCCAACAGACCCACAA |
|  | 2 | - | 999 | 1021 | a1a7f2b2_1_RIGHT_6 | GCAAAGCCCAGAAGACCCACAA |
|  | 2 | - | 999 | 1021 | a1a7f2b2_1_RIGHT_7 | GCGAACCCCAAAAGACCCACAA |
| 2 | 1 | + | 881 | 906 | a1a7f2b2_2_LEFT_0 | GGATATGTAATTGGGAGTTGGGGCA |
|  | 1 | + | 881 | 906 | a1a7f2b2_2_LEFT_1 | GGATATGTAATTGGGTGTTGGGGCA |
|  | 1 | + | 880 | 906 | a1a7f2b2_2_LEFT_2 | GGGATATGTAATTGGAAGTTGGGGCA |
|  | 1 | + | 880 | 906 | a1a7f2b2_2_LEFT_3 | GGGATATGTCATTGGAAGTTGGGGTA |
|  | 1 | + | 880 | 906 | a1a7f2b2_2_LEFT_4 | GGGCTATGTCATTGGATGTTATGGGT |
|  | 1 | + | 880 | 906 | a1a7f2b2_2_LEFT_5 | GGGGTATGTAATTGGAAGTTGGGGTA |
|  | 1 | + | 880 | 906 | a1a7f2b2_2_LEFT_6 | GGGTTACATAATTGGAAGTTGGGGAA |
|  | 1 | + | 880 | 906 | a1a7f2b2_2_LEFT_7 | GGGTTATGTAATTGGAAGTTGGGGGT |
|  | 1 | + | 881 | 906 | a1a7f2b2_2_LEFT_8 | GGTTATGTTATTGGCAGTTGGGGAT |
|  | 1 | + | 876 | 906 | a1a7f2b2_2_LEFT_9 | TCATGGGATATATAATTGGAAGTTGGGGTA |
|  | 1 | + | 879 | 906 | a1a7f2b2_2_LEFT_10 | TGGGATATGTAATTGGAAGTTGGGGTA |
|  | 1 | + | 879 | 906 | a1a7f2b2_2_LEFT_11 | TGGGATATGTAATTGGATGTTGGGGTA |
|  | 1 | - | 1388 | 1408 | a1a7f2b2_2_RIGHT_0 | AGGATCCAGTTGGCAGCACA |
|  | 1 | - | 1388 | 1411 | a1a7f2b2_2_RIGHT_1 | CGAAGGATCCAGTTGGCAGTACA |
|  | 1 | - | 1388 | 1410 | a1a7f2b2_2_RIGHT_2 | GCAGGATCCAGTTGGCAGTACA |
|  | 1 | - | 1388 | 1412 | a1a7f2b2_2_RIGHT_3 | GCGAAGAATCCAGTTGGCAGTACA |
| 3 | 2 | + | 1173 | 1195 | a1a7f2b2_3_LEFT_0 | TCTGCCAAGTGTTTGCTGATGC |
|  | 2 | + | 1175 | 1195 | a1a7f2b2_3_LEFT_1 | TGCCAAGTGTTTGCTGACGC |
|  | 2 | + | 1173 | 1195 | a1a7f2b2_3_LEFT_2 | TGTGCCAAGTGTTTGCTGATGC |
|  | 2 | - | 1681 | 1703 | a1a7f2b2_3_RIGHT_0 | CCTCAAGGTCGGTCGTTGACAT |
|  | 2 | - | 1681 | 1704 | a1a7f2b2_3_RIGHT_1 | GCCTCAAGGTCGGTTGTTGACAT |
| 4 | 1 | + | 1579 | 1600 | a1a7f2b2_4_LEFT_0 | TGTGCACTTCGCTTCACCTCT |
|  | 1 | - | 2111 | 2136 | a1a7f2b2_4_RIGHT_0 | ATCTTCCAAATTAACACCCACCCAG |
|  | 1 | - | 2111 | 2136 | a1a7f2b2_4_RIGHT_1 | ATCTTCCAAATTTACACCCACCCAG |
|  | 1 | - | 2111 | 2134 | a1a7f2b2_4_RIGHT_2 | CGTCCAAATTACTTCCCACCCAG |
|  | 1 | - | 2111 | 2134 | a1a7f2b2_4_RIGHT_3 | CTTCCAAATTACCACCCACCCAG |
|  | 1 | - | 2111 | 2137 | a1a7f2b2_4_RIGHT_4 | GATCTTCCAAATTACTTCCCACCCAG |
|  | 1 | - | 2111 | 2138 | a1a7f2b2_4_RIGHT_5 | GGATCTTCCAAATTATTACCCACCCAG |
|  | 1 | - | 2111 | 2136 | a1a7f2b2_4_RIGHT_6 | GTCTTCCAAATTACTTCCCACCCAG |
|  | 1 | - | 2111 | 2135 | a1a7f2b2_4_RIGHT_7 | TCTTCCAAATTACCTCCCACCCAG |
| 5 | 2 | + | 2020 | 2042 | a1a7f2b2_5_LEFT_0 | AGGCCTTAGAGTCTCCGGAACA |
|  | 2 | + | 2019 | 2042 | a1a7f2b2_5_LEFT_1 | GAAGCCTTAGAGTCTCCTGAGCA |
|  | 2 | + | 2019 | 2042 | a1a7f2b2_5_LEFT_2 | GATGCCTTAGAGTCTCCTGAGCA |
|  | 2 | + | 2018 | 2042 | a1a7f2b2_5_LEFT_3 | GGATGCCTTAGAATCTCCTGAGCA |
|  | 2 | - | 2535 | 2564 | a1a7f2b2_5_RIGHT_0 | CCTGCAAATGAATGTTAGGAAAAGAAGGA |
|  | 2 | - | 2535 | 2564 | a1a7f2b2_5_RIGHT_1 | CCTGTAAATGAATGTCAGGAAAAGAAGGA |
|  | 2 | - | 2535 | 2564 | a1a7f2b2_5_RIGHT_2 | CCTGTAAATGAATGTTAGGAAAGGAGGGA |
|  | 2 | - | 2535 | 2566 | a1a7f2b2_5_RIGHT_3 | CTCTTGTAAATGAATCTTAGGAAAGGAAGGA |
|  | 2 | - | 2535 | 2567 | a1a7f2b2_5_RIGHT_4 | CTTGGTGTAAATGTATATTAGGAAAAGAGGGT |
|  | 2 | - | 2535 | 2561 | a1a7f2b2_5_RIGHT_5 | GCAAATGAATGTGAGGAAAGGAGGGA |
|  | 2 | - | 2535 | 2564 | a1a7f2b2_5_RIGHT_6 | GGTGCAAATGAATATCAGGAAAAGATGGA |
|  | 2 | - | 2535 | 2568 | a1a7f2b2_5_RIGHT_7 | TCTTGGTGTAAATGTATATTAGGAAAAGAAGGT |
|  | 2 | - | 2535 | 2568 | a1a7f2b2_5_RIGHT_8 | TCTTGGTGTAAATGTATATTAGGAAAAGATGGT |
|  | 2 | - | 2535 | 2565 | a1a7f2b2_5_RIGHT_9 | TCTTTCAAATGAATGTGAGGAAAAGAAGGA |
|  | 2 | - | 2535 | 2562 | a1a7f2b2_5_RIGHT_10 | TGCAAATGAATGTCAGGAAAAGAAGGA |
|  | 2 | - | 2535 | 2562 | a1a7f2b2_5_RIGHT_11 | TGCAAATGAATGTTAGGAAAAGAGGGA |
|  | 2 | - | 2535 | 2562 | a1a7f2b2_5_RIGHT_12 | TGTAAATGAATGTGAGGAAAGGAGGGA |
|  | 2 | - | 2535 | 2563 | a1a7f2b2_5_RIGHT_13 | TTTCAAATGAATGTGAGGAAAAGAGGGA |
| 6 | 1 | + | 2412 | 2435 | a1a7f2b2_6_LEFT_0 | CCGCGTCGCAGAAGATCTAAATC |
|  | 1 | + | 2413 | 2435 | a1a7f2b2_6_LEFT_1 | CGCGTCGCAGAAGATCTCAATC |
|  | 1 | - | 2949 | 2970 | a1a7f2b2_6_RIGHT_0 | AAGGGTCCTTGTTGGGGTTGA |
|  | 1 | - | 2949 | 2970 | a1a7f2b2_6_RIGHT_1 | AGTTGTCCTTGTGCGGGTTGA |
|  | 1 | - | 2949 | 2971 | a1a7f2b2_6_RIGHT_2 | CAGTGGTCCTTGATGGGGTTGA |
|  | 1 | - | 2949 | 2972 | a1a7f2b2_6_RIGHT_3 | CCAGGTGTCCTTGTTGGGATTGA |
|  | 1 | - | 2949 | 2972 | a1a7f2b2_6_RIGHT_4 | CCAGTGATCCTTGTTGGGGTTGA |
|  | 1 | - | 2949 | 2972 | a1a7f2b2_6_RIGHT_5 | CCAGTTGTCCTTGTGTGGGTTGA |
|  | 1 | - | 2949 | 2973 | a1a7f2b2_6_RIGHT_6 | GCCAATGATCCTTGTTGGGGTTGA |
|  | 1 | - | 2949 | 2973 | a1a7f2b2_6_RIGHT_7 | GCCAATTGTCCTTGTGTGGATTGA |
|  | 1 | - | 2949 | 2973 | a1a7f2b2_6_RIGHT_8 | GCCATTGATCCTTGTTGGGGTTGA |
|  | 1 | - | 2949 | 2974 | a1a7f2b2_6_RIGHT_9 | GGCCAATTGTCCTTGTTTTTGTTGA |
|  | 1 | - | 2949 | 2973 | a1a7f2b2_6_RIGHT_10 | TCCAGTGGTCTTTGTTGGGATTGT |
| 7 | 2 | + | 2814 | 2838 | a1a7f2b2_7_LEFT_0 | CGGGTCACCATATTCTTGGGAACA |
|  | 2 | + | 2815 | 2838 | a1a7f2b2_7_LEFT_1 | GGGTCACCATATTCCTGGGAACA |
|  | 2 | + | 2814 | 2838 | a1a7f2b2_7_LEFT_2 | TGGGTCACCATATTCTTGGGAACA |
|  | 2 | + | 2814 | 2838 | a1a7f2b2_7_LEFT_3 | TGGGTCACCTTATTCTTGGGAACA |
|  | 2 | - | 248 | 272 | a1a7f2b2_7_RIGHT_0 | AGAGAAGTCCACCACGAGTCTAGA |

**Supplementary table 6: final 6 amplicon scheme (hbv/600/v2.1.0/)**

Numbering based on X02763 reference genome

| Amplicon  **hbv/600/v2.1.0** | Pool | Strand | Start location | End location | Primer name | Primer sequence |
| --- | --- | --- | --- | --- | --- | --- |
| 0 | 1 | + | 97 | 123 | f3d7635a_0_LEFT_0 | AATACTGCCTCTGCCATATCATCAAC |
|  | 1 | + | 98 | 123 | f3d7635a_0_LEFT_1 | ACACTGCCTCTTCCATATCGTCAAT |
|  | 1 | + | 97 | 123 | f3d7635a_0_LEFT_2 | ACTACTGCCTCTCACATATCGTCAAT |
|  | 1 | + | 97 | 123 | f3d7635a_0_LEFT_3 | ACTACTGCCTCTCACTTATCGTCAAT |
|  | 1 | + | 97 | 123 | f3d7635a_0_LEFT_4 | ACTATTGCCTCTCACATCTCGTCAAT |
|  | 1 | + | 100 | 123 | f3d7635a_0_LEFT_5 | ACTGCCTCACCCATATCGTCAAT |
|  | 1 | + | 100 | 123 | f3d7635a_0_LEFT_6 | ACTGCCTCACTCATCTCGTCAAT |
|  | 1 | + | 100 | 123 | f3d7635a_0_LEFT_7 | ACTGCCTCTCACATCTCGTCAAT |
|  | 1 | + | 100 | 123 | f3d7635a_0_LEFT_8 | ACTGCCTCTGCCATATCGTCAAC |
|  | 1 | + | 95 | 123 | f3d7635a_0_LEFT_9 | AGAATACTGCCTCTTCCATATCGTCAAT |
|  | 1 | + | 95 | 123 | f3d7635a_0_LEFT_10 | AGAATACTGTCTCAGCCATATCGTCAAT |
|  | 1 | + | 95 | 123 | f3d7635a_0_LEFT_11 | AGAATACTGTCTCTGCCATATCGTCAAT |
|  | 1 | + | 95 | 123 | f3d7635a_0_LEFT_12 | CAAATATTGCCTCTCACATCTCGTCAAT |
|  | 1 | + | 99 | 123 | f3d7635a_0_LEFT_13 | CACTGCCTCTCCCATATCGTCAAT |
|  | 1 | + | 95 | 123 | f3d7635a_0_LEFT_14 | CGACTACTGTCTCTCACATATCGTCAAT |
|  | 1 | + | 95 | 123 | f3d7635a_0_LEFT_15 | CGACTATTGCCTCTCTCACATCATCAAT |
|  | 1 | + | 98 | 123 | f3d7635a_0_LEFT_16 | CTACTGCCTCTCCCATATCGTCAAT |
|  | 1 | + | 98 | 123 | f3d7635a_0_LEFT_17 | CTACTGCCTCTCCCTTATCGTCAAT |
|  | 1 | + | 96 | 123 | f3d7635a_0_LEFT_18 | GAATATTGCCTCTCACATCTCGTCAAT |
|  | 1 | + | 96 | 123 | f3d7635a_0_LEFT_19 | GACTACTGTCTCTCCCATATCGTCAAT |
|  | 1 | + | 102 | 123 | f3d7635a_0_LEFT_20 | TGCCTCTCCCACATCGTCAAT |
|  | 1 | - | 737 | 760 | f3d7635a_0_RIGHT_0 | CCCCCAATACCACATCATCCACA |
|  | 1 | - | 737 | 761 | f3d7635a_0_RIGHT_1 | GCCCCCAAAACCACATCATCCATA |
|  | 1 | - | 737 | 761 | f3d7635a_0_RIGHT_2 | GCCCCCAATACCACATCATCCATA |
|  | 1 | - | 737 | 761 | f3d7635a_0_RIGHT_3 | GCCCCCAATACCAGATCATCCATA |
|  | 1 | - | 737 | 761 | f3d7635a_0_RIGHT_4 | GCCCCCAGTACCACATCATCCATA |
|  | 1 | - | 737 | 762 | f3d7635a_0_RIGHT_5 | GGCCCCCAAAACCATATCATCCATA |
|  | 1 | - | 737 | 762 | f3d7635a_0_RIGHT_6 | GGCCCCCAATACCAAATCATCCATA |
|  | 1 | - | 737 | 762 | f3d7635a_0_RIGHT_7 | GGCCCCCAATACCACATCATCAATA |
|  | 1 | - | 737 | 762 | f3d7635a_0_RIGHT_8 | GGCCCCCAATACCATATCATCCATA |
| 1 | 2 | + | 588 | 614 | f3d7635a_1_LEFT_0 | AAATTGCACTTGTATTCCCATCCCAT |
|  | 2 | + | 590 | 614 | f3d7635a_1_LEFT_1 | ACTGCACTTGTATTCCCATCCCAT |
|  | 2 | + | 592 | 614 | f3d7635a_1_LEFT_2 | TGCACCTGTATTCCCATCCCAT |
|  | 2 | - | 1168 | 1188 | f3d7635a_1_RIGHT_0 | AGCAAACACTTGGCACAGGC |
|  | 2 | - | 1168 | 1191 | f3d7635a_1_RIGHT_1 | ATCAGCAAACACTTGGCAGAGAC |
|  | 2 | - | 1168 | 1192 | f3d7635a_1_RIGHT_2 | CATCAGCAAACACTTGGCAAAGAC |
|  | 2 | - | 1168 | 1192 | f3d7635a_1_RIGHT_3 | CGTCAGCAAACACTTGGCAAAGAC |
|  | 2 | - | 1168 | 1192 | f3d7635a_1_RIGHT_4 | CGTCAGCAAACACTTGGCATAGAC |
|  | 2 | - | 1168 | 1193 | f3d7635a_1_RIGHT_5 | GCATCAGCAAACACTTGGCATAGAC |
|  | 2 | - | 1168 | 1191 | f3d7635a_1_RIGHT_6 | GTCAGCAAACACTTGGCAGAGAC |
|  | 2 | - | 1168 | 1190 | f3d7635a_1_RIGHT_7 | TCAGCAAACACTTGGCAAAGGC |
|  | 2 | - | 1168 | 1190 | f3d7635a_1_RIGHT_8 | TCAGCAAACACTTGGCACAGAC |
| 2 | 1 | + | 1081 | 1106 | f3d7635a_2_LEFT_0 | AACAGGCTTTTACTTTTTCGCCAAC |
|  | 1 | + | 1082 | 1106 | f3d7635a_2_LEFT_1 | ACAGGCTTTTACTTTCTCGCCAAC |
|  | 1 | + | 1084 | 1106 | f3d7635a_2_LEFT_2 | AGGCTTTCACTTTCTCGCCAAC |
|  | 1 | + | 1083 | 1106 | f3d7635a_2_LEFT_3 | CAGGCTTTCACTTTTTCGCCAAC |
|  | 1 | + | 1083 | 1106 | f3d7635a_2_LEFT_4 | CAGGCTTTTGTCTTTTCGCCAAC |
|  | 1 | + | 1082 | 1106 | f3d7635a_2_LEFT_5 | GCAAGCTTTCACTTTCTCGCCAAC |
|  | 1 | + | 1082 | 1106 | f3d7635a_2_LEFT_6 | GCAGGCTTTTACTTTCTCGCCAAC |
|  | 1 | + | 1083 | 1106 | de464cad_2_LEFT_7 | CAGGCTTTTGTTTTCTCGCCAAC |
|  | 1 | - | 1692 | 1720 | f3d7635a_2_RIGHT_0 | ACACAGTCTTTGATGTATTCTTCGATCC |
|  | 1 | - | 1692 | 1718 | f3d7635a_2_RIGHT_1 | ACAGTCTTTGAAGTAAGCCTCAAGGT |
|  | 1 | - | 1692 | 1718 | f3d7635a_2_RIGHT_2 | ACAGTCTTTGAAGTATGCCTCAAGGT |
|  | 1 | - | 1692 | 1716 | f3d7635a_2_RIGHT_3 | AGTCTTTGAAGTACGCCTCAAGGT |
|  | 1 | - | 1692 | 1716 | f3d7635a_2_RIGHT_4 | AGTCTTTGAAGTAGGCCTCAAGGT |
|  | 1 | + | 1692 | 1719 | de464cad_2_RIGHT_1 | CACAGTCCTTGAAGTATTTCTCCATCC |
|  | 1 | + | 1692 | 1720 | de464cad_2_RIGHT_2 | ACACAGTCTTTGATGTATTCCTCAATCC |
|  | 1 | + | 1692 | 1718 | de464cad_2_RIGHT_3 | ACAGTCCTTGAAGTATTTCTCCACCC |
| 3 | 2 | + | 1640 | 1666 | f3d7635a_3_LEFT_0 | CCAACAGTCTTACATAAGCGGACTCT |
|  | 2 | + | 1641 | 1666 | f3d7635a_3_LEFT_1 | CCAAGGTCTTACATAAGCGGACTCT |
|  | 2 | + | 1641 | 1666 | f3d7635a_3_LEFT_2 | CCAAGGTCTTGCATAAGAGGACTCT |
|  | 2 | + | 1640 | 1666 | f3d7635a_3_LEFT_3 | CCCAAGGTCTTACATAAGAGGACTCT |
|  | 2 | + | 1639 | 1666 | f3d7635a_3_LEFT_4 | GCCAACAGTCTTACATAAGAGGACTCT |
|  | 2 | + | 1639 | 1666 | f3d7635a_3_LEFT_5 | GCCCAAGGTCTTACATAAAAGGACTCT |
|  | 2 | + | 1639 | 1666 | f3d7635a_3_LEFT_6 | GCCCAAGGTCTTACATAAGAGGACTAT |
|  | 2 | + | 1639 | 1666 | f3d7635a_3_LEFT_7 | GCCCAAGGTCTTATATAAGAGGACTCT |
|  | 2 | + | 1639 | 1666 | f3d7635a_3_LEFT_8 | GCCTAAGGTCTTACATAAGAGGACTCT |
|  | 2 | + | 1638 | 1666 | de464cad_3_LEFT_9 | TGCCAAAGCAGTTATATAAGAGGACTCT |
|  | 2 | + | 1639 | 1666 | de464cad_3_LEFT_10 | GCCAAGGCAGTTATATAAGAGGACTCT |
|  | 2 | - | 2299 | 2325 | f3d7635a_3_RIGHT_0 | ATTGATAAGATAGGGGCATTTGGTGG |
|  | 2 | - | 2299 | 2326 | f3d7635a_3_RIGHT_1 | CGTTGATAAGATAGGGGCATTTTGTGG |
|  | 2 | - | 2299 | 2323 | f3d7635a_3_RIGHT_2 | GGATAGGATAGGGGCATTTGGTGG |
|  | 2 | - | 2299 | 2325 | f3d7635a_3_RIGHT_3 | GTTGATAAGATAGGGGCATTTGCTGG |
|  | 2 | - | 2299 | 2325 | f3d7635a_3_RIGHT_4 | GTTGATAAGATAGGGGCATTTGGAGG |
|  | 2 | - | 2299 | 2325 | f3d7635a_3_RIGHT_5 | GTTGATAAGATAGGGGCATTTGGTGG |
|  | 2 | - | 2299 | 2323 | f3d7635a_3_RIGHT_6 | TGATAGGATAGGGGCATTTGGTGG |
|  | 2 | - | 2299 | 2324 | f3d7635a_3_RIGHT_7 | TGGATAAGATAGGGGCATTTGGTGG |
|  | 2 | - | 2299 | 2326 | f3d7635a_3_RIGHT_8 | TGTTGATAAGATAGGGGCATTAGGTGG |
|  | 2 | - | 2299 | 2326 | f3d7635a_3_RIGHT_9 | TGTTGATAAGATAGGGGCATTTGATGG |
|  | 2 | - | 2299 | 2326 | f3d7635a_3_RIGHT_10 | TGTTGATAAGATAGGGGCATTTGTTGG |
|  | 2 | - | 2299 | 2326 | f3d7635a_3_RIGHT_11 | TGTTGATAAGATAGGGGCATTTTGTGG |
| 4 | 1 | + | 2204 | 2232 | f3d7635a_4_LEFT_0 | GGTTCCACATTTCCTGTCTTACTTTTGG |
|  | 1 | + | 2204 | 2232 | f3d7635a_4_LEFT_1 | GGTTTCACATCTCCTGTCTTACTTTTGG |
|  | 1 | + | 2204 | 2232 | f3d7635a_4_LEFT_2 | GGTTTCACATTTCCTGTCTTACCTTTGG |
|  | 1 | + | 2204 | 2232 | f3d7635a_4_LEFT_3 | GGTTTCACATTTCCTGTCTTACTTTCGG |
|  | 1 | + | 2204 | 2232 | f3d7635a_4_LEFT_4 | GGTTTCACATTTCCTGTCTTACTTTTGG |
|  | 1 | + | 2204 | 2232 | f3d7635a_4_LEFT_5 | GGTTTCACATTTCTTGTCTCACTTTTGG |
|  | 1 | + | 2202 | 2232 | f3d7635a_4_LEFT_6 | GTGGTTTCATATATCTTGCCTTACTTTTGG |
|  | 1 | + | 2202 | 2232 | f3d7635a_4_LEFT_7 | GTGGTTTCATATTTCCTGTCTTACTTTTGG |
|  | 1 | + | 2205 | 2232 | f3d7635a_4_LEFT_8 | GTTTCACACTTCTTGTCTCACTTTTGG |
|  | 1 | + | 2205 | 2232 | f3d7635a_4_LEFT_9 | GTTTCACATTTCCTGCCTTACTTTTGG |
|  | 1 | + | 2205 | 2232 | f3d7635a_4_LEFT_10 | GTTTCACATTTCCTGTCTCACTTTTGG |
|  | 1 | + | 2205 | 2232 | f3d7635a_4_LEFT_11 | GTTTCACGTTTCTTGTCTCACTTTTGG |
|  | 1 | + | 2203 | 2232 | f3d7635a_4_LEFT_12 | TGGTTTCACATTTCTTGTCTTACTTTTGG |
|  | 1 | + | 2203 | 2232 | f3d7635a_4_LEFT_13 | TGGTTTCATATTTCTTGCCTTACTTTTGG |
|  | 1 | + | 2204 | 2232 | de464cad_4_LEFT_14 | GGTTTCATATTTCCTGCCTTACATTTGG |
|  | 1 | + | 2205 | 2232 | de464cad_4_LEFT_15 | GTTTCACATTTCCTGCCTTACATTTGG |
|  | 1 | - | 2834 | 2855 | f3d7635a_4_RIGHT_0 | ATGCCGTAGCTCTTGTTCCCA |
|  | 1 | - | 2834 | 2857 | f3d7635a_4_RIGHT_1 | CCATGCTGTAGCTCTTGTTCCCA |
|  | 1 | - | 2834 | 2857 | f3d7635a_4_RIGHT_2 | CCATGCTGTAGCTTTTGTTCCCA |
|  | 1 | - | 2834 | 2858 | f3d7635a_4_RIGHT_3 | CCCATGATGTAGCTCTTGTTCCCA |
|  | 1 | - | 2834 | 2858 | f3d7635a_4_RIGHT_4 | CCCATGCTGTAGATCTTGTTCCCA |
|  | 1 | - | 2834 | 2858 | f3d7635a_4_RIGHT_5 | CCCATGCTGTATCTCTTGTTCCCA |
|  | 1 | - | 2834 | 2858 | f3d7635a_4_RIGHT_6 | GGTTTGCTGTAGCTCTTGTTCCCA |
| 5 | 2 | + | 2768 | 2794 | f3d7635a_5_LEFT_0 | AAGGCGGGCATTCTATATAAGAGAGA |
|  | 2 | + | 2769 | 2794 | f3d7635a_5_LEFT_1 | AGGCGGGCATCTTATATAAAAGGGA |
|  | 2 | + | 2769 | 2794 | f3d7635a_5_LEFT_2 | AGGCTGGGATCCTATATAAGAGGGA |
|  | 2 | + | 2764 | 2794 | f3d7635a_5_LEFT_3 | ATGGAAGGCGGGTATATTATATAAGAGAGA |
|  | 2 | + | 2760 | 2794 | f3d7635a_5_LEFT_4 | CTTTATGGAAAGCAGGAATTCTATATAAGAGAGA |
|  | 2 | + | 2767 | 2794 | f3d7635a_5_LEFT_5 | GAAGGCTGGGATTCTATATAAGAGGGA |
|  | 2 | + | 2766 | 2794 | f3d7635a_5_LEFT_6 | GGAAGGCGGGAATCTTATATAAGAGAGA |
|  | 2 | + | 2766 | 2794 | f3d7635a_5_LEFT_7 | GGAAGGCGGGCATCTTATATAAAAGAGA |
|  | 2 | + | 2766 | 2794 | f3d7635a_5_LEFT_8 | GGAAGGCGGGGATCTTATATAAAAGAGA |
|  | 2 | + | 2766 | 2794 | f3d7635a_5_LEFT_9 | GGAAGGCGGGTATCTTATATAAGAGAGA |
|  | 2 | + | 2766 | 2794 | f3d7635a_5_LEFT_10 | GGAAGGCGGGTGTATTATATAAGAGAGA |
|  | 2 | + | 2766 | 2794 | f3d7635a_5_LEFT_11 | GGAAGGCTGGCATTCTATATAAGAGAGA |
|  | 2 | + | 2766 | 2794 | f3d7635a_5_LEFT_12 | GGAAGGCTGGTATTCTATATAAGAGGGA |
|  | 2 | + | 2764 | 2794 | f3d7635a_5_LEFT_13 | GTGGAAGGCTGGTATTCTATATAAGAGAGA |
|  | 2 | + | 2765 | 2794 | f3d7635a_5_LEFT_14 | TGGAAAGCTGGCATTCTATATAAGAGAGA |
|  | 2 | + | 2765 | 2794 | f3d7635a_5_LEFT_15 | TGGAAGGCGGGAATTTTATATAAGAGAGA |
|  | 2 | + | 2765 | 2794 | f3d7635a_5_LEFT_16 | TGGAAGGCGGGTATCTTATATAAAAGAGA |
|  | 2 | + | 2765 | 2794 | f3d7635a_5_LEFT_17 | TGGAAGGCGGGTATTTTATATAAGAGAGA |
|  | 2 | + | 2765 | 2794 | f3d7635a_5_LEFT_18 | TGGAAGGCTGGCATTCTATATAAAAGAGA |
|  | 2 | + | 2765 | 2794 | f3d7635a_5_LEFT_19 | TGGAAGGCTGGCATTTTATATAAGAGAGA |
|  | 2 | + | 2763 | 2794 | f3d7635a_5_LEFT_20 | TGTGGAAGGCTGGTATTTTATATAAGAGAGA |
|  | 2 | + | 2763 | 2794 | f3d7635a_5_LEFT_21 | TTTGGAAGGCGGGTATTTTATATAAAAGAGA |
|  | 2 | + | 2766 | 2794 | de464cad_5_LEFT_22 | GGAAGGCGGGTATTCTATATAAGAGAGA |
|  | 2 | + | 2765 | 2794 | de464cad_5_LEFT_23 | TGGAAGGCAGGAATTCTATATAAGAGAGA |
|  | 2 | - | 225 | 249 | f3d7635a_5_RIGHT_0 | AGACTCTGCGGTATTGTGAGGATT |
|  | 2 | - | 225 | 254 | f3d7635a_5_RIGHT_1 | AGTCTAGACTCTTTGGTATTGTGAGGATT |
|  | 2 | - | 225 | 253 | f3d7635a_5_RIGHT_2 | GTCTAGACTCTGTGGTATTGTGAGGATT |
|  | 2 | - | 225 | 253 | f3d7635a_5_RIGHT_3 | GTCTAGACTCTTCGGTATTGTGAGGATT |

**Supplementary text**

**Determining HBV drug resistance and immune escape from sequences using HBV WGS**

We were able to call polymorphisms at sites of previously reported resistance associated mutations (RAMs) and vaccine escape mutations (VEMs) as defined by geno2pheno[38]. Polymorphisms were detected in RT and HBsAg regions. In the HBsAg region, mutations associated with HBsAg escape from vaccines and detection were identified for example D144E (ULIDS-0610). All samples with polymorphisms had only been sequenced with a single method, HEP-TILE Nanopore.
